# History of Surgery in Kentucky

**Published:** 1852-07

**Authors:** 


### HISTORY OF SURGERY IN KENTUCKY.

We invite the attention of our Kentucky readers to the subjoined circular. And are there not among the many physicians who have gone from Kentucky, some who could communicate valuable facts touching the surgical history of their native State? All who are in possession of information on this subject will, no doubt, take pleasure in contributing to make the proposed history complete.

*To the Physicians of Kentucky.*—The undersigned, chairman of the committee on surgery, of the Kentucky Medical Society, begs leave, in compliance with the duties assigned to him, to call the attention of his professional brethren, in all parts of the State, to the subject, and to request them to send to him, on or before the 15th of August next, all such facts and observations within their knowledge as may be calculated to illustrate the history and practice of surgery, from the earliest settlement of the country to the present moment. It is the sincere desire of the undersigned not to overlook or pass unnoticed the claims of any one, and he therefore earnestly invites the cooperation of all.

Louisville, June 19, 1852. S. D. GROSS, M. D.

